# Bacterial community structure and effects of picornavirus infection on the anterior nares microbiome in early childhood

**DOI:** 10.1186/s12866-018-1372-8

**Published:** 2019-01-07

**Authors:** Mahrrouz Caputo, Beate Zoch-Lesniak, André Karch, Marius Vital, Frederic Meyer, Frank Klawonn, Armin Baillot, Dietmar H. Pieper, Rafael T. Mikolajczyk

**Affiliations:** 1 Department of Epidemiology, Helmholtz Centre for Infection Research, Inhoffenstraße 7, 38124 Braunschweig, Germany; 2 PhD Programme “Epidemiology”, Braunschweig, Germany; 3 PhD Programme “Epidemiology”, Hannover, Germany; 4 German Centre for Infection Research (DZIF), Hannover-Braunschweig site, Inhoffenstraße 7, 38124 Braunschweig, Germany; 5 Institute for Epidemiology and Social Medicine, University of Münster, Domagkstraße 3, 48149 Münster, Germany; 6 Microbial Interactions and Processes Research Group, Helmholtz Centre for Infection Research, Inhoffenstraße 7, 38124 Braunschweig, Germany; 7 Microbial Communication Research Group, Helmholtz Centre for Infection Research, Inhoffenstraße 7, 38124 Braunschweig, Germany; 8 Biostatistics Research Group, Helmholtz Centre for Infection Research, Inhoffenstraße 7, 38124 Braunschweig, Germany; 9 Institute of Information Engineering, Ostfalia University, Salzdahlumer Str. 46/48, 38302 Wolfenbüttel, Germany; 10 Governmental Institute of Public Health of Lower Saxony, Roesebeckstraße 4-6, 30449 Hannover, Germany; 11 Institute for Medical Epidemiology, Biometrics, and Informatics (IMEBI), Medical Faculty of the Martin Luther University Halle-Wittenberg, Magdeburger Str. 8, 06110 Halle (Saale), Germany

**Keywords:** Anterior nares, Early childhood, Picornavirus infection, Temporal diversity, Temporal dynamics

## Abstract

**Background:**

Little is known regarding the nasal microbiome in early childhood and the impact of respiratory infection on the infants’ nasal microbial composition. Here we investigated the temporal dynamics and diversity of the bacterial composition in the anterior nares in children attending daycare centers.

**Results:**

For our investigation, we considered 76 parental-taken nasal swabs of 26 children (aged 13 to 36 months) collected over a study period of 3 months. Overall, there was no significant age-specific effect or seasonal shift in the nasal bacterial community structure. In a sub-sample of 14 healthy children the relative abundance of individual taxa as well as the overall diversity did not reveal relevant changes, indicating a stable community structure over the entire study period. Moreover, the nasal bacterial profiles clustered subject-specific with Bray-Curtis similarities being elevated in intra-subject calculations compared to between-subject calculations. The remaining subset of 12 children provided samples taken during picornavirus infection (PVI) and either before or after a PVI. We detected an association between the relative abundance of members of the genus *Streptococcus* and PV when comparing both (i) samples taken during PVI with samples out of 14 healthy children and (ii) samples taken during PVI with samples taken after PVI within the same individual. In addition, the diversity was higher during PVI than after infection.

**Conclusions:**

Our findings suggest that a personalized structure of the nasal bacterial community is established already in early childhood and could be detected over a timeframe of 3 months. Studies following infants over a longer time with frequent swab sampling would allow investigating whether certain parameter of the bacterial community, such as the temporal variability, could be related to viral infection.

**Electronic supplementary material:**

The online version of this article (10.1186/s12866-018-1372-8) contains supplementary material, which is available to authorized users.

## Background

The respiratory microbiome plays an important role in preventing diseases by inhibiting the colonization of incoming pathogens and priming the immune defense [1, 2]. Since anterior nares are permanently exposed to the outside environment and represent an initial entry point of potential viral and bacterial pathogens, the role of the anterior nares’ microbial community in disease development is of great interest, particularly in infancy when the burden of respiratory infections is highest. Two recent longitudinal studies have investigated the dynamics in bacterial composition of the healthy nostrils in early childhood [3, 4], describing a high heterogeneity across individuals, and indicating age and season as most influencing factors for the microbial diversity. Distinct bacterial community structures in the anterior nares of children and adults were reported [5, 6]. Accordingly, the anterior nares’ microbiome seems to undergo dramatic changes with age. Still, further investigations are required to assess its development during infancy.

Disturbance of the bacterial homeostasis, including that of the nares, might enhance the overgrowing of pathogens and lead to a higher susceptibility to diseases [1]. Possible dysbioses of the nasal microbiome were investigated in children with pre-existing diseases, such as cystic fibrosis [7]. In addition, an interaction of specific pathogens and the nasal microbial flora was studied, but predominately in cross-sectional studies [8–10]. By now, only one study focused on the impact of respiratory infections on the global bacterial community structure in the anterior nares in healthy children during the first year of life [11]. The aim of our study was to assess the temporal dynamics and diversity of the nasal bacterial community in children attending daycare centers and to study the effects of picornaviruses (PV), the most common viral respiratory pathogen identified in our samples during symptomatic infections, on the nasal community composition.

## Methods

### Study population and collection of samples

This analysis is a secondary data analysis of nasal swabs from a feasibility study for a birth cohort study focusing on infections, the microbiome, and the development of the immune system in childhood [12]. In brief, 75 families with children aged 13 to 36 months were recruited in daycare centers in Braunschweig, Germany, between November 2013 and March 2014. Parents were asked to answer a baseline questionnaire (covering information about e.g. sex and age), to keep a daily symptom diary on respiratory symptoms, and to take monthly nasal swabs (routine samples) on assigned days (independent from the occurrence of symptoms) over a period of 3 months (see Additional file 1 in reference [12]). Parents received the required equipment as well as detailed instructions for the use of Liquid Amies Elution anterior nasal swabs (ESwab, Copan, Italy). After sampling, the parents transferred the swab back into the tube, which was filled with 1 ml Amies medium. They were asked to store the samples in a 4 °C fridge until mailing to the study center. The time between swab sampling and final storage at the study center was in median 2 days (interquartile range (IQR): 1 to 2 days). For the analysis of the bacterial community structure in healthy children, we included only children who displayed no symptoms of respiratory infection on any of the three swab collection days, roughly 1 month apart. Besides collecting routine samples, parents were also asked to take one nasal swab during a respiratory infection [12]. To assess the impact of viral infection on the bacterial composition, we selected picornaviruses, the most common viral pathogen associated with respiratory infections in our study population [12]. Samples and questionnaire data were collected after obtaining written informed consent from all guardians and parents as previously described [12].

### DNA preparation and amplicon sequencing

After arrival of the samples at the study center, nasal swabs were vortexed for 20 s to allow further dissemination from the swab into the medium. The samples were aliquoted and stored at − 80 °C until analysis. For investigating the bacterial community structure the swab was used for DNA extraction using FastDNA Spin Kit for Soil (MP Biomedicals, Solon, OH, USA) based on the manufacturers’ instruction. The hypervariable region V1/V2 of the 16S rRNA gene was subsequently amplified and sequenced on an Illumina MiSeq (2 × 250 bp, Illumina, Hayward, California, USA) as previously described [6].

### Bioinformatic analysis

Bioinformatic processing was performed as previously described [6]. Raw reads were merged with the Ribosomal Database Project (RDP) assembler. Overall, 2,351,406 reads were obtained with a mean of 30,939 (± 13,453 standard deviation) reads per sample. Sequences were aligned within MOTHUR (gotoh algorithm using the SILVA reference database) and subjected to pre-clustering (diffs = 2, [13]) yielding phylotypes that were filtered for an average abundance of ≥0.001% and a sequence length ≥ 250 bp before analysis. All samples were re-sampled to equal the smallest read size of 12,761 reads using the phyloseq package [14]. Phylotypes were assigned to a taxonomic affiliation based on the naïve Bayesian classification [15] with a threshold of 80% followed by manual curation [6].

### Detection of viral pathogens

The identification of viral pathogens was performed at the Governmental Institute of Public Health of Lower Saxony (NLGA) using 200 μl of the sample. The samples were tested for six different viruses (adenovirus, metapneumovirus, picornavirus, respiratory syncytial virus, influenza A and B virus) as previously described [12]. Given the small sample size and the low number of laboratory confirmed infections, we decided to focus only on the most frequently detected virus, which was picornavirus (PV) (including human rhino- and enteroviruses) (Additional file 1: Figure S1). The detection of PV was performed via reverse transcriptase polymerase chain reaction (RT-PCR) with the help of a Rhino- & Enterovirus-Kit/Cc r-gene (BioMérieux/ARGENE) using a Light-Cycler (Roche).

### Statistical analysis

A dataset comprising the relative abundance of all phylotypes for each sample with aggregated levels for phylum and genera was created using PRIMER 6 software (v.6.1.6, PRIMER-E, Plymouth Marine Laboratory, Plymouth, UK). Genera with a mean relative abundance across all samples below 1% as well as unclassified bacteria were summarized as “Others”. Anterior nare community diversity was analyzed using total phylotype number, Simpson index (1-Lambda), Pielou’s evenness (J’) and Shannon diversity (H′ loge) using PRIMER 6 software [16]. For the detection of differences in the relative abundance between infected and healthy children, samples from healthy children were randomly selected by generating a random number sorted in ascending order. For each child only one single sample was selected. Statistical testing was conducted applying Wilcoxon signed-rank tests and Mann-Whitney-U-Tests using Stata IC for Windows, version 12 (StataCorp, College Station, TX). The findings were subsequently corrected for multiple testing by the Benjamini-Hochberg procedure (also known as the false discovery rate approach) [17].

To assess similarity between microbial community profiles of each child the Bray-Curtis similarity algorithm (with square root transformation) on the phylotype level was used and visualized in non-metric multidimensional scaling (nMDS). Significant differences between the predefined groups were analyzed using One-way Analysis of similarities (ANOSIM) with 999 permutations with corresponding Global-R statistics. *R*-values indicate the degree of separation between groups; values closer to 1 indicate clear distinct groups [16]. For investigating variation within subjects, the index of multivariate dispersion (IMD) and similarity percentages (SIMPER) was calculated based on the Bray-Curtis similarity algorithm with square root transformation on phylotype level. Higher values of IMD indicate a higher heterogeneity of the bacterial profile over time. These investigations were performed with PRIMER v6 software.

In order to assess whether the intra-individual distances in the healthy children were smaller than random distances, we used a permutation-based stochastic simulation with 10,000 runs via R (Version 3.2.). For this, samples of the healthy children were randomly exchanged (10,000 times) across all children and the mean of the mean Bray-Curtis similarities of these runs was calculated. These findings were compared with the actual Bray-Curtis similarity of the bacterial profiles in the samples of the healthy children.

## Results

### Study population and effects of age and season on bacterial community structure

We included 14 children (aged 19 to 33 months) in this study who displayed no symptoms of respiratory infection (“healthy children”) on any of the three swab collection days (42 swabs, Table 1) which were roughly 1 month apart (days between sampling: 28, IQR: 27 to 31). To assess the impact of viral infections on bacterial community structures, we selected 12 additional children (aged 13 to 34 months) with a positive PCR-result for PV and reported respiratory symptoms in the symptom diary (minimum symptoms were cough or a runny/blocked nose) (“infected children”). These 12 infected children provided samples taken during a PVI and samples taken before and/or after a PVI (34 swabs, Table 1).

**Table 1:** Baseline characteristics of healthy children (*n* = 14) and infected children (*n* = 12)

Variables	Healthy children	Infected children
	(*n* = 14)	(*n* = 12)
Sex of children, *n* (%)		
Male	7 (50%)	9 (75%)
Female	7 (50%)	3 (25%)
Age of children in months, median (IQR)	29 (19; 30)	18 (14; 33)
Number of provided nasal swabs by time of collection	First month (14)	Before PVI (9)^a,b^
	Second month (14)	During PVI (12)
	Third month (14)	After PVI (13)^b,c^
Days between swab sampling, median (IQR)	28 (27; 31)	29 (28; 31)

*IQR* Interquartile range, *PVI* Picornavirus infection

^a^Three children provided two samples and one child provided one sample before PVI

^b^Two children provided samples before and after PVI

^c^Five children provided two samples and one child provided one sample after PVI

For investigating age-specific effects on the bacterial community structure, all 76 samples of 26 children included in the current study were categorized into three groups depending on the age at collection day (group 1: 13 to 19 months of age, *n* = 22; group 2: 20 to 29 months, *n* = 23; group 3: 30 to 36 months, *n* = 31). The microbial community profile of each child was analyzed using the Bray-Curtis similarity algorithm (with square root transformation) on the phylotype level and visualized in a nMDS. The nMDS plot showed no clear clustering by age-group (ANOSIM: *R* = 0.02; *p*-value = 0.210; Additional file 2: Figure S2). In addition, the bacterial profiles were grouped by season of sampling time (winter (*n* = 39) vs. spring (*n* = 37)), but no evidence for seasonal clustering was observed (ANOSIM: *R* = 0.01; *p*-value = 0.202; Additional file 2: Figure S2).

### Bacterial community structure across the anterior nares in healthy children

**Fig. 1:**
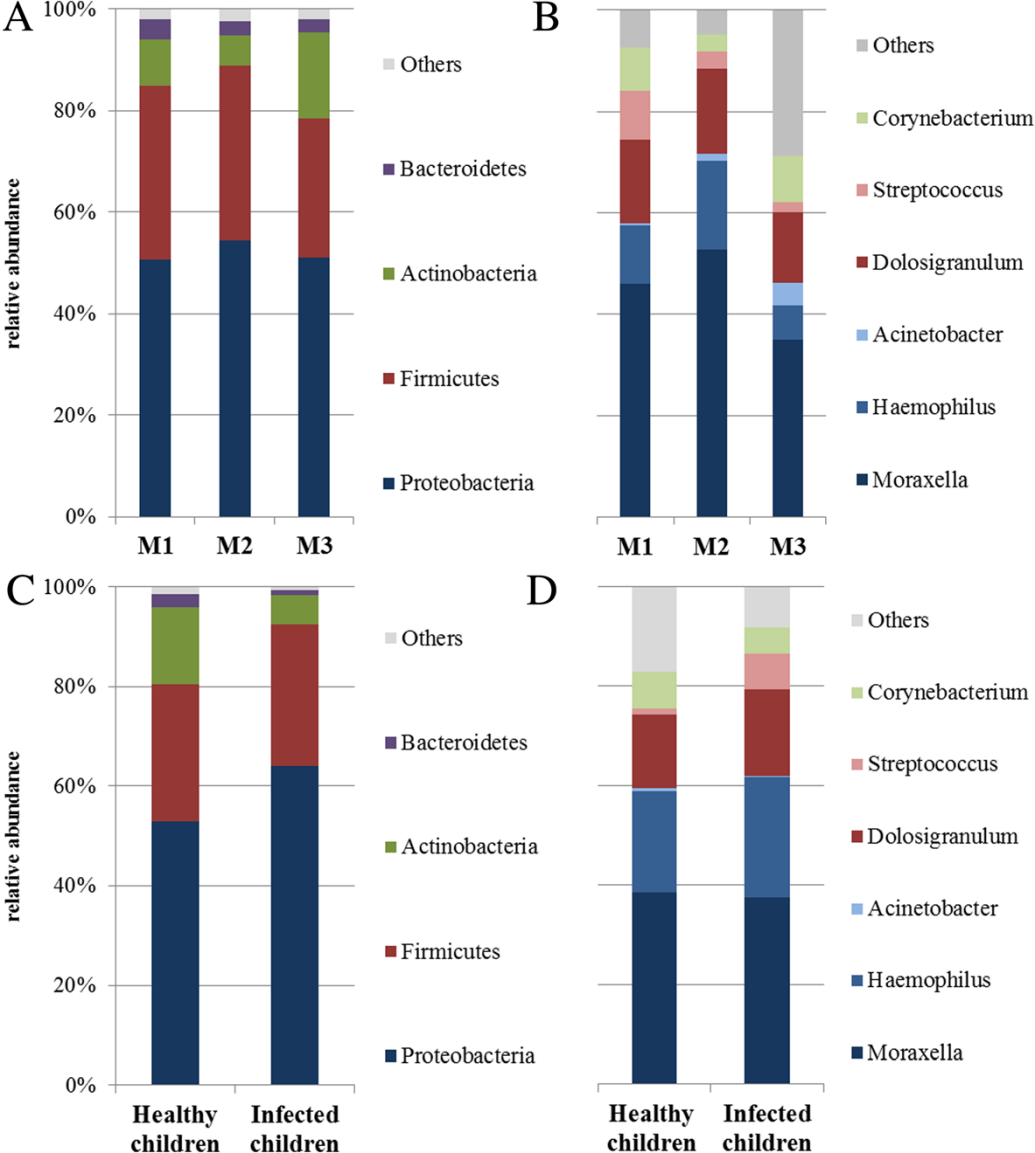
Average relative abundance of bacterial phyla and genera in the anterior nares. **a** phyla and **b** genera of 14 healthy children at three different collection days roughly 1 month apart (M1, *n* = 14; M2, *n* = 14; M3 *n* = 14). Comparison of the mean relative abundance of (**c**) phyla and (**d**) genera in children infected with picornavirus (*n* = 12) and healthy children (*n* = 12)

**Fig. 2:**
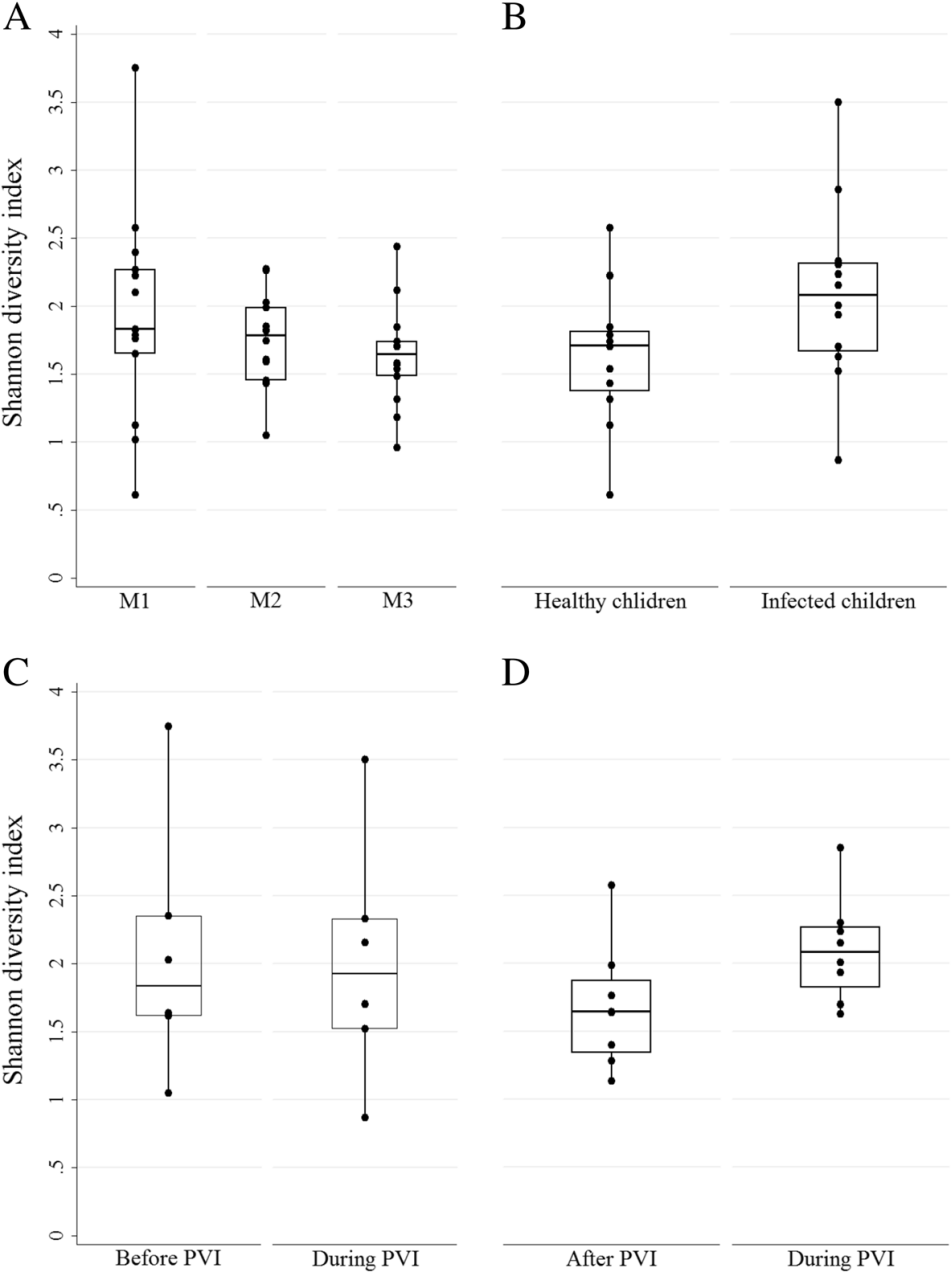
Alpha-diversity of the anterior nares bacterial community by Shannon diversity. **a** Shannon diversity of 14 healthy children on three different collection days, roughly 1 month apart (M1, *n* = 14; M2, *n* = 14; M3 *n* = 14). **b** Shannon diversity was compared between samples of healthy children (*n* = 12) and samples of additional 12 infected children taken during picornavirus infection (PVI) (*n* = 12). **c** Shannon diversity from samples taken before and during PVI within the same infected children (*n* = 6). **d** Shannon diversity from samples taken during and after PVI within the same infected children (*n* = 8). The Shannon diversity of each child is represented by one dot

**Fig. 3:**
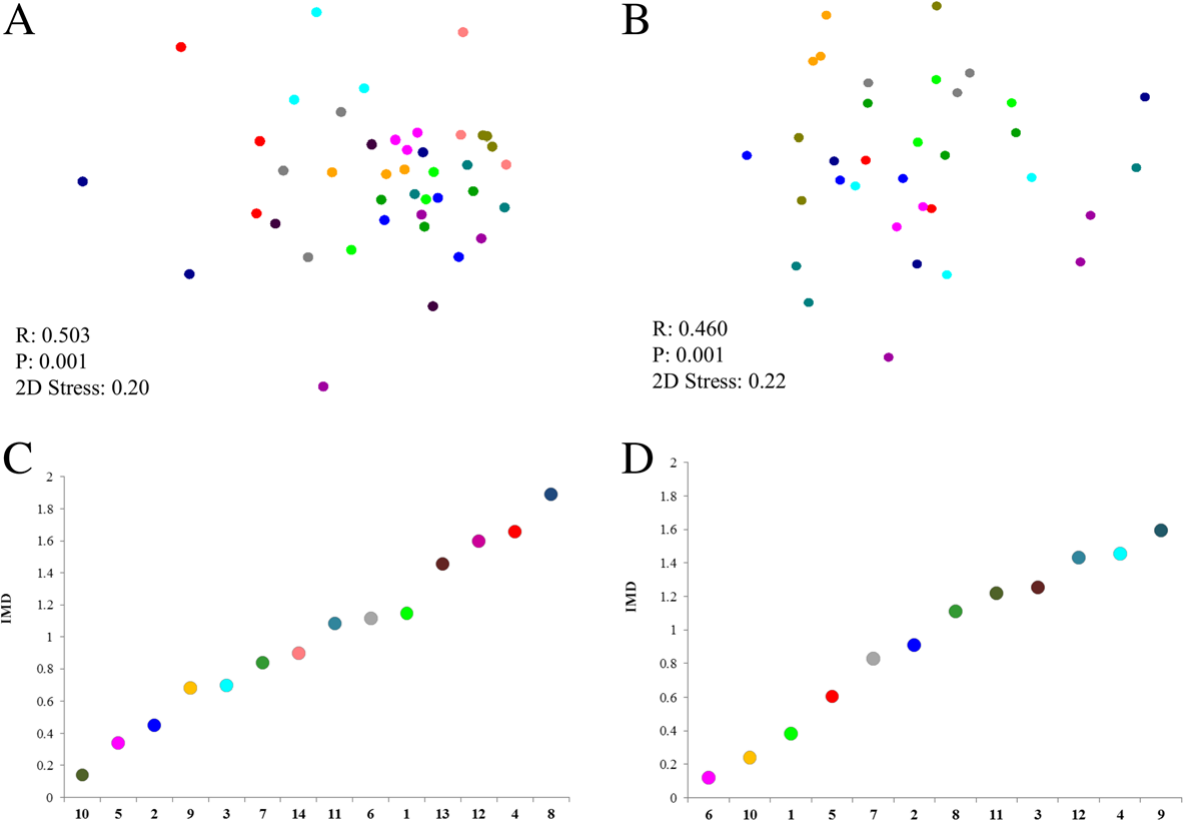
Non-metric multidimensional scaling (nMDS) of the global nasal bacterial community structure and index of multivariate dispersion (IMD). **a** nMDS and **c** IMD in 14 healthy children between the bacterial profiles of three sampling times for each healthy child (*n* = 42). **b** nMDS and **d** IMD of 12 infected children, where each child is represented by one color with either two or three samples per child (during picornavirus infection (PVI) *n* = 12, before PVI *n* = 9, after PVI *n* = 13). The color assignment for the IMD corresponds to the ones in the nMDS plot of each child. R: *R*-value, indicates the degree of separation between groups; P: *p*-value; 2D Stress: indicates the two-dimensional stress level on the plot

**Fig. 4:**
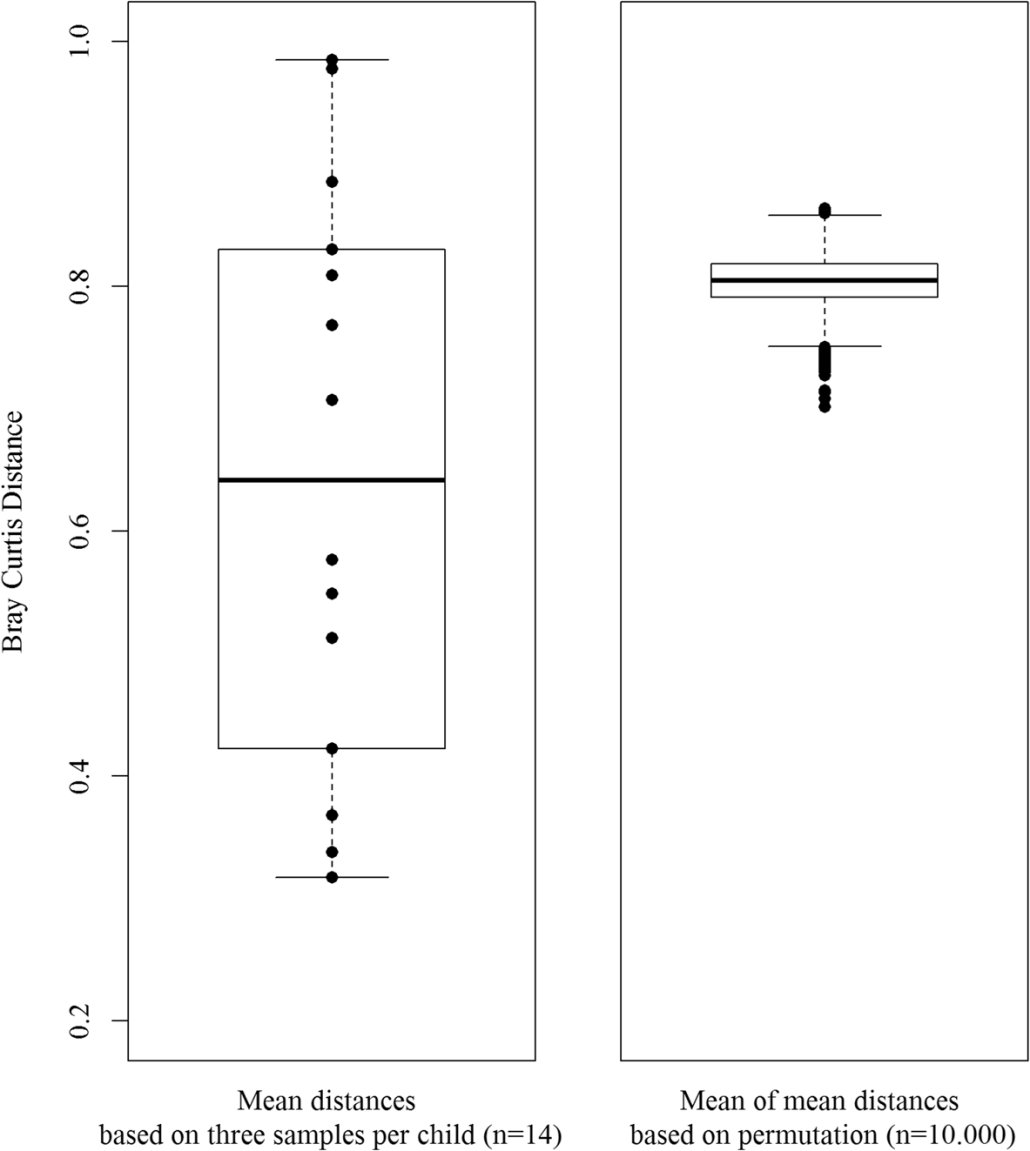
Comparison of the average distance between bacterial profiles of three different collection days roughly 1 month apart of 14 healthy children (left) with mean of mean distances based on permutations using the Bray-Curtis similarity algorithm (right)

The bacterial community structures of the anterior nares across all 42 collected samples were dominated by *Proteobacteria* (52.0%), *Firmicutes* (33.0%), and *Actinobacteria* (10.8%) (Fig. 1a). On the genus level, *Moraxella* showed the highest mean relative abundance of 44.5% followed by *Dolosigranulum* (15.7%) (Fig. 1b and Additional file 3: Figure S3). To investigate changes in the community structure over the period of 3 months we performed a pairwise comparison of the average relative abundance on the genus level of samples taken during the first (M1), second (M2) and third month (M3) using the Wilcoxon signed-rank test. Distinct differences were only detected in “Others” (representing detected bacteria with a relative abundance < 1% and unclassified bacteria) between M1 and M3 (*p*-value = 0.030; after correction for multiple testing not significant [17]). The anterior nares community diversity was analyzed by Shannon diversity (H’), Simpson diversity index (1-Lambda) and Pielou’s evenness on phylotype level, indicating also no significant difference between the three bacterial profiles (M1, M2 and M3) using Wilcoxon signed-rank tests (Fig. 2a and Additional file 4: Figure S4). With respect to the total phylotype lower absolute numbers were detectable at M3 (120.5, IQR: 82.0 to 147.8) compared to M1 (157.0, IQR: 125.8 to 125.8; *p*-value = 0.030) and M2 (160.1, IQR: 150.8 to 181.5; *p*-value = 0.013). The bacterial community structure of each child over time was compared by nMDS plot displaying a high heterogeneity among the 14 children (Fig. 3a; ANOSIM: *R* = 0.50; *p*-value = 0.001) and a clustering on intra-individual level for some children. To further investigate whether the bacterial community shows subject-specific signatures, we conducted a permutation-based stochastic simulation. The mean Bray-Curtis similarity of three samples per child ranged between 0.32 and 0.99, with an overall mean of 0.65 (Fig. 4). When the samples were randomly exchanged across the children based on permutation, the overall mean was 0.80 (based on 10,000 simulations). The IMD ranged between 0.14 (Child 10, with a corresponding SIMPER of 63.4%) and 1.89 (Child 8, SIMPER 11.9%) (Fig. 3c). Thus, child 8 represented a rather instable, heterogeneous community structure and child 10 a stable and much more homogeneous community structure within the observed period of 3 months. In addition, we could show significantly higher Bray-Curtis similarity within-subjects than between-subjects (Additional file 5: Table S1).

### Effects of picornavirus infection on bacterial community across anterior nares

A higher relative abundance of members of the genus *Streptococcus* was observed in infected children (7.2%) compared to healthy children (1.3%) (*p*-value = 0.038; after correction for multiple testing not significant [17]) (Fig. 1d and Additional file 6: Figure S5).

To assess longitudinal effects of PVI we considered samples taken during PVI as well as samples taken before (*n* = 9) and after (*n* = 13) a PVI within the 12 infected children (Additional file 7: Figure S6). An overall difference in bacterial profiles between the three sampling times (before, during and after PVI) could be excluded (ANOSIM: *R* = 0.0; *p*-value = 0.936). The IMDs ranged between 0.12 (Child 6, SIMPER 59.9%) and 1.60 (Child 9, SIMPER 20.1%) (Fig. 3b and 3d).

We wanted to assess whether the abundance of bacterial genera are related with the occurrence of PVI. For this purpose, we compared the microbial composition approximately 1 month before a PVI with the bacterial composition during a PVI. In all, five children provided samples taken approximately 1 month before PVI and one child were sampled 2 months before a PVI (Additional file 7: Figure S6). We found no significant differences (Additional file 8: Figure S7, left). Longitudinal effects of PVI were investigated in children providing both, samples taken during as well as after a PVI. This applies to eight children, where a higher relative abundance of *Streptococcus* (5.2%) during a PVI was observed as 1 month after a PVI (1.5%) (Additional file 8: Figure S7, right) (*p*-value = 0.025; after adjusting for multiple-testing both differences were not significant [17]).

### Effects of picornavirus infection on anterior nares’ community diversity

The comparison of Shannon diversity (H′) on phylotype level between bacterial communities of 12 healthy (2.09, IQR: 1.64 to 2.32) and 12 infected children (1.63, IQR: 1.34 to 1.83) showed no significant difference (*p*-value = 0.119, Mann-Whitney-U-Test; Fig. 2b). In addition, Simpson diversity index (1-Lambda), Pielou’s evenness (J’) and richness on phylotype level did not significantly differ in the presence of PV (Additional file 4: Figure S4).

Investigating longitudinal effects of PVI on the diversity within the 12 infected children, we only detected differences comparing samples taken during and samples taken approximately 1 month after PVI within the same individuals (*n* = 8). The presence of PV was associated with a significantly higher Shannon diversity (2.10, IQR: 1.76 to 2.28; *p*-value = 0.012; Fig. 2d) as well as a higher Pielou’s evenness (0.43, IQR: 0.37 to 0.44; *p*-value = 0.012) and a higher Simpson diversity index (1-Lambda) (0.76, IQR: 0.69 to 0.84; *p*-value = 0.036; Additional file 9: Figure S8).

## Discussion

We analyzed the temporal dynamics and diversity as well as longitudinal effects of PVI on the anterior nares’ bacterial community in early childhood. Analyzing 76 nasal swabs of 26 children, we found no age-specific or seasonal clustering of the bacterial structure. Even though PVI did not lead to a global change of the bacterial composition, we found an association between PVI and the members of *Streptococcus* and longitudinal effects on the alpha-diversity.

In a previous study, it was reported that developmental age is a major factor affecting the dynamics of the nasal microbiome during the first year of life [3, 4]. Since our study population consisted of children of different ages (13 to 36 months of age), we investigated whether the bacterial community structure was associated with age. However, we found no significant effects between the pre-defined age-groups (13 to 19, 20 to 29, and 30 to 36 months of age). One reason could be that the high inter-individual heterogeneity superimposed an actual age-specific relation. Furthermore, it is possible that in the investigated age-range of 13 to 36 months the bacterial structure did not significantly differ by age because it had already developed a stable individual signature at this age. In previous studies, distinct age-specific differences were detected comparing the nasal bacterial structure during childhood and adulthood [5, 6]. In addition, we also investigated effects of season on the microbial composition and found no differences between samples taken in winter and spring. Previous studies described different bacterial profiles between summer and winter months in children [3], and a shift in community profile of the anterior nares in adults between the months February and March [18]. The fact that we could not observe distinct seasonal bacterial profiles could be due to the comparatively mild winter in Lower Saxony in the recruitment year, with a small temperature difference between winter and spring (Additional file 10: Table S2).

The relative abundance of phyla in the anterior nares of the 14 healthy children were in line with findings from a previous study among children from Gabon [6]. To assess the temporal bacterial stability in healthy children we considered various approaches. The visualization of the bacterial profiles via nMDS depicted a high heterogeneity between children; however, based on IMD and SIMPER we found clustering on intra-individual level for a study period of 3 months for some children. This observation was confirmed by findings from the random permutation showing that on average there was some clustering effect within individuals. The observed variation of the temporal bacterial stability between the children has already been reported in adults [18]. In addition, we could show significantly higher Bray-Curtis similarity within-subjects than between-subjects, which is in line with findings of Mika et al. [3]. Our data suggests that a personalized structure of the nasal microbiome establishes already in early childhood, despite microorganisms inhabiting the anterior nares are permanently facing changing conditions of the external environment.

To our knowledge, we are one of the first groups investigating the effect of a viral infection on the bacterial community of the anterior nostril. Within our study we did not observe a global effect of PVI on the bacterial community structure. However, the presence of PV was associated with a higher relative abundance of members of the genus *Streptococcus*. This observed association is in line with previous studies. In healthy children attending daycare, a positive association between *Streptococcus pneumoniae* colonization density and viral load of PV in the nasopharynx was described [10]. In addition, a positive association has previously been shown between *S. pneumoniae* and the abundance of human rhinovirus (HRV) in nasopharyngeal samples of 6 to 24 months old healthy children [19]. HRV belongs to PV and represents the most common cause for upper respiratory tract infection [20]. In children aged 4 to 7 years with and without asthma, Kloepfer et al. detected a greater quantity of bacterial species, including *S. pneumoniae*, during an HRV infection [21]. HRV is known to affect the cell adhesion molecules and therefore the bacterial adherence in human nasal epithelial cells [1, 22]. In presence of HRV, fibronectin expression is increased which is associated with better adhesion of *S. pneumoniae* [1, 22]. The observed comparatively lower abundance of *Streptococcus* after PVI might be due to a decrease in fibronectin protein expression that leads to a reduction of *Streptococcus* adhesion. However, this hypothesis has to be further tested.

Besides changes of the relative abundance, we also investigated effects on the bacterial diversity. We observed no significant difference in Shannon diversity between healthy and infected children. In a previous study on adults’ nasopharynx microbiota a significantly lower alpha-diversity (Shannon diversity index) during HRV infection was found compared to not infected individuals [23]. However, the study was based on ten subjects (seven infected and three non-infected adults), who have been sampled several times, making the study susceptible for potential outliers. Since for this investigation, we had a small sample size, potential effects of a PVI on the bacterial diversity could have been masked by high inter-individual variation. Additionally, we considered samples taken within the same individuals. We found a higher Shannon diversity after a PVI than during a PVI. This is in contrast to the findings of Korten et al., reporting a lower Shannon diversity of the nasal microbiome during symptomatic HRV infection compared to the diversity of samples taken 3 weeks after HRV infection [11].

In our study, we were able to investigate the bacterial composition in a longitudinal manner considering both, bacterial composition and viral pathogens. Further, we compared the anterior bacterial community structure in healthy as well as in infected children. Nonetheless, there are various limitations in our study. The sample size for our investigation is relatively small, making our findings more susceptible to distortion by individual outliers. While it might have been of interest to use a broader approach in our analysis, we restricted ourselves to 16S rRNA based approach at genera level and performed PCR based identification of selected viral pathogens rather than realizing whole virome analysis. We investigated longitudinal effects of viral infections approximately 1 month after PVI. Over this period, short time changes of the nasal bacterial composition could have already been reversed to the pre-infection state. A more frequent and dense sampling (e.g. on daily or weekly base) after respiratory infections would allow a much more precise time-depended analysis but would require a different study design. Since our study was carried out during winter/spring season, we did not have data on the bacterial composition in summer/autumn, thus restricting the possibility of analyzing seasonal effects on the nasal microbiome. In addition, there might be different host and environmental factors affecting the individual bacterial profile, also during respiratory infections such as medication or co-infections. Especially regarding the long-term investigations on the individual nasal bacterial composition after respiratory infections, the use of drugs such as nasal spray or antibiotics is highly relevant. To better understand the effects of various environmental factors on the temporal dynamics and diversity of the nasal bacterial community additional longitudinal studies are needed with a higher number of study participants covering continuous data collection on these factors and information on respiratory symptoms, as planned for the prospective cohort study LöwenKIDS (ClinicalTrials.gov identifier: NCT02654210).

## Conclusion

Overall, our findings revealed no changes of the anterior nares bacterial profile of healthy children visiting daycare centers aged 13 to 36 months over a timeframe of 3 months. Although the stability within children varied, our data showed high subject-specific signatures suggesting a stable community structure within the study period. This adds evidence towards a personalized and stable bacterial composition in the anterior nares that is established already in early childhood. While PVI did not lead to overall changes of the bacterial ecosystem of the nostril, we detected an association between PVI and *Streptococcus* carriage. Whether certain parameters of bacterial communities such as temporal variability are related to viral infections has to be investigated within studies that follow infants over a longer time. This will allow disentangling the complex relationships between the nasal microbiota and susceptibility to infection.

## Additional files

Additional file 1: **Figure S1.** Frequency of detected viral pathogens. (PDF 46 kb)

Additional file 2: **Figure S2.** Non-metric multidimensional scaling (nMDS) plot comparing the global bacterial community structure of the anterior nares by season and age across 26 children providing 76 samples. (PDF 83 kb)

Additional file 3: **Figure S3.** Average relative abundance of selected genera in the anterior nares’ bacterial community over three different collection days of nasal swabs across 14 healthy children. (PDF 105 kb)

Additional file 4: **Figure S4.** Anterior nares’ community diversity indicated by total phylotype number, Simpson index (1-Lambda), and Pielou’s evenness (J’). (PDF 233 kb)

Additional file 5: **Table S1.** Comparison of within-subject and between-subject average similarity percentage of the anterior nares’ bacterial profiles across 14 healthy children. (PDF 17 kb)

Additional file 6: **Figure S5.** Relative abundance of selected genera in the anterior nares bacterial community of 12 healthy children and 12 additional children with picornavirus infection (PVI). (PDF 127 kb)

Additional file 7: **Figure S6.** Number and timing of nasal swab collection from 12 children with picornavirus infection (PVI). (PDF 54 kb)

Additional file 8: **Figure S7.** Average relative abundance of genera in the anterior nares’ bacterial communities of samples taken during and either before or after a picornavirus infection (PVI) within the same children. (PDF 98 kb)

Additional file 9: **Figure S8.** Anterior nares’ community diversity in infected children indicated by total phylotype number, Simpson index (1-Lambda), and Pielou’s evenness (J’). (PDF 229 kb)

Additional file 10: **Table S2.** Seasonal mean air temperature in Lower Saxony for winter and spring between 2010 and 2016. (PDF 31 kb)

## Abbreviations

ANOSIM: Analysis of similarities
HRV: Human rhinovirus
IMD: Index of multivariate dispersion
IQR: Interquartile range
nMDS: Non-metric multidimensional scaling
PV: Picornavirus
PVI: Picornavirus infection
RDP: Ribosomal Database Project
SIMPER: Similarity percentages
