# Supplementary material for: Bacterial community structure and effects of picornavirus infection on the anterior nares microbiome in early childhood
Source: BMC Microbiol. 2019 Jan 7;19:1. doi: 10.1186/s12866-018-1372-8 (PMC6322332; doi:10.1186/s12866-018-1372-8)
Supplement: Supplementary file 1 — Figure S1. Frequency of detected viral pathogens. (PDF 46 kb) [file 12866_2018_1372_MOESM1_ESM.pdf]

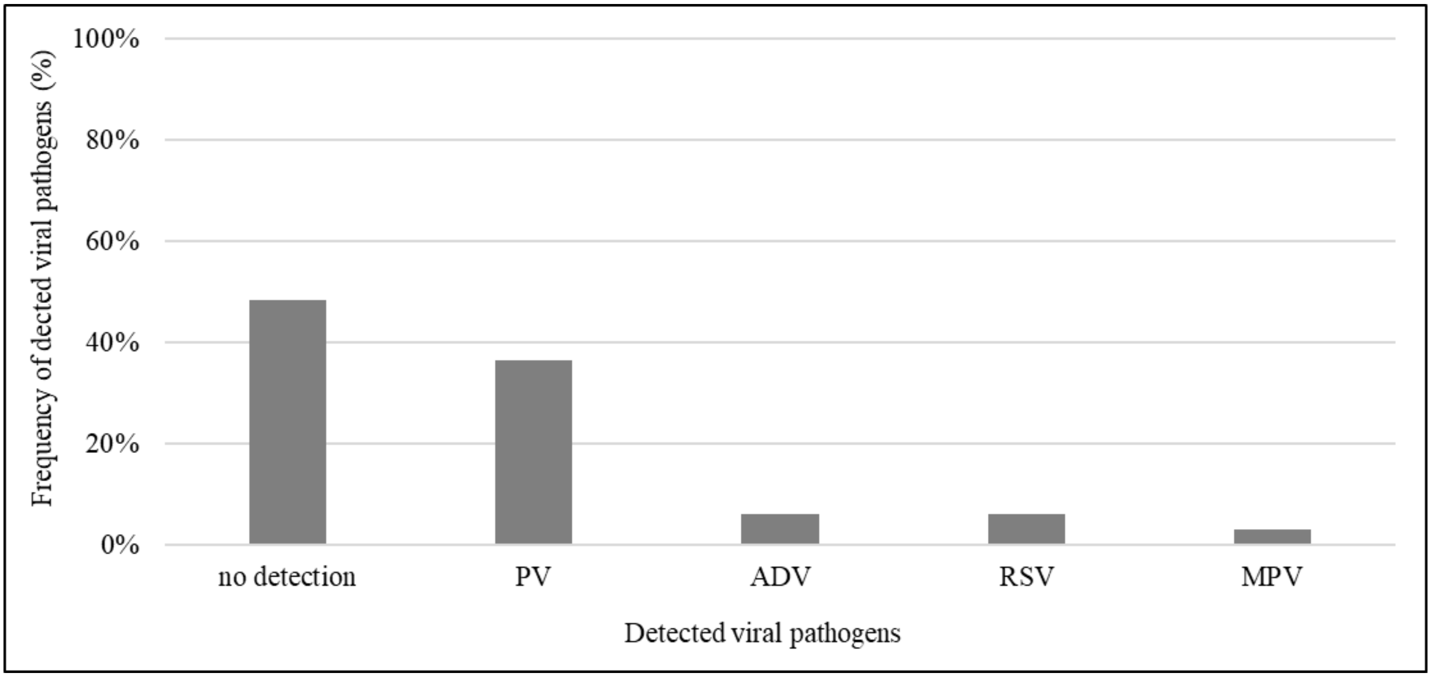

Figure S1. Frequency of detected viral pathogens.
We included 30 samples tested for six different viral pathogens. In twelve samples picornavirus was detected (PV, 36%), in two samples adenovirus (ADV, 6%), in two samples respiratory syncytial virus (RSV, 6%), and in one sample metapneumovirus (MPV, 3%). In 16 samples none of the six viral pathogens could be detected (no detection, 48%). Influenza A or B virus was not found.
