# Supplementary material for: Bacterial community structure and effects of picornavirus infection on the anterior nares microbiome in early childhood
Source: BMC Microbiol. 2019 Jan 7;19:1. doi: 10.1186/s12866-018-1372-8 (PMC6322332; doi:10.1186/s12866-018-1372-8)
Supplement: Supplementary file 2 — Figure S2. Non-metric multidimensional scaling (nMDS) plot comparing the global bacterial community structure of the anterior nares by season and age across 26 children providing 76 samples. (PDF 83 kb) [file 12866_2018_1372_MOESM2_ESM.pdf]

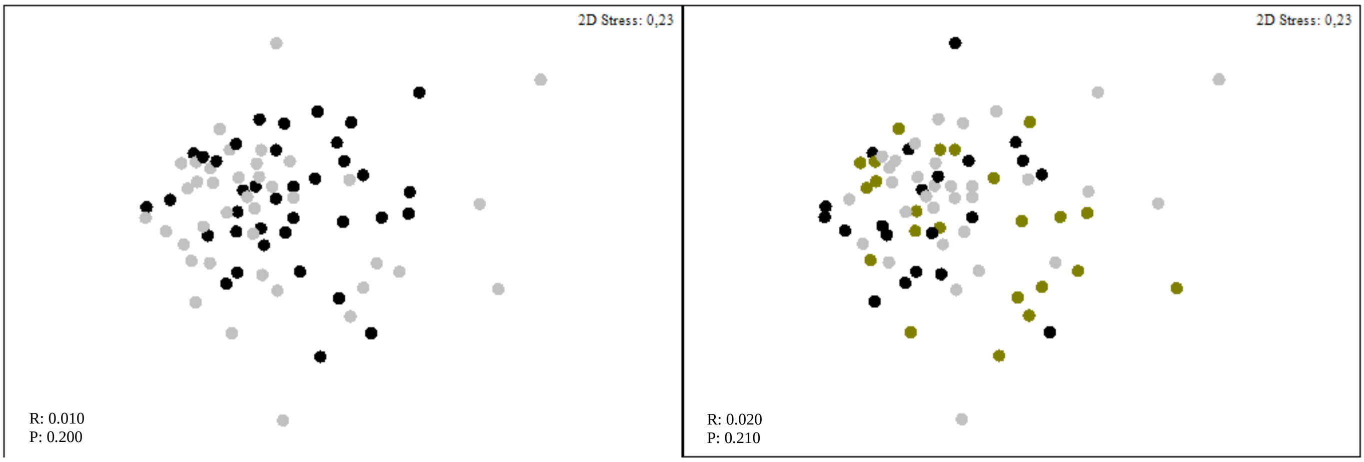

Figure S2. Non-metric multidimensional scaling (nMDS) plot comparing the global bacterial community structure of the anterior nares by season and age across 26 children providing 76 samples.
Community structures across 26 children with 39 samples taken during winter season (●) and 37 samples during spring season (●) (left); Community structures across 26 children with samples taken at 13 to 19 months of age ((●) n=22), 20 to 29 ((●) n=23), and 30 to 36 months of age at collection day ((●) n=31) (right); One-way Analysis of similarities (ANOSIM) with 999 permutations was calculated with corresponding Global-R statistics and P-value to assess similarity between bacterial profiles (see inset); Analyses were performed with PRIMER v6 software.
