# Supplementary material for: Bacterial community structure and effects of picornavirus infection on the anterior nares microbiome in early childhood
Source: BMC Microbiol. 2019 Jan 7;19:1. doi: 10.1186/s12866-018-1372-8 (PMC6322332; doi:10.1186/s12866-018-1372-8)
Supplement: Supplementary file 3 — Figure S3. Average relative abundance of selected genera in the anterior nares’ bacterial community over three different collection days of nasal swabs across 14 healthy children. (PDF 105 kb) [file 12866_2018_1372_MOESM3_ESM.pdf]

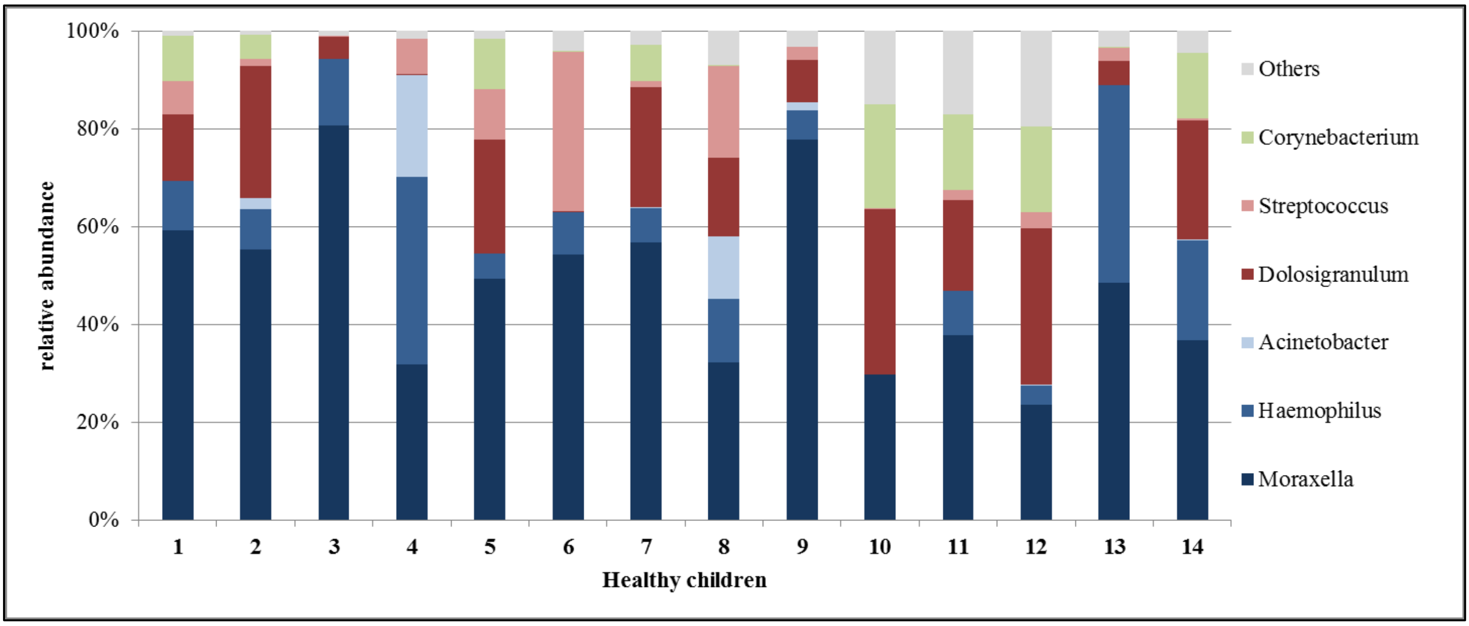

Figure S3. Average relative abundance of selected genera in the anterior nares´ bacterial community over three different collection days of nasal swabs across 14 healthy children.
Bacterial community structure in 14 healthy children, who displayed no symptoms of respiratory infection on any of the three collection days of nasal swabs; Bacteria with an average relative abundance below 1% and unclassified bacteria were summarized as "Others".
