# Supplementary material for: Bacterial community structure and effects of picornavirus infection on the anterior nares microbiome in early childhood
Source: BMC Microbiol. 2019 Jan 7;19:1. doi: 10.1186/s12866-018-1372-8 (PMC6322332; doi:10.1186/s12866-018-1372-8)
Supplement: Supplementary file 5 — Table S1. Comparison of within-subject and between-subject average similarity percentage of the anterior nares’ bacterial profiles across 14 healthy children. (PDF 17 kb) [file 12866_2018_1372_MOESM5_ESM.pdf]

Table S1. Comparison of within-subject and between-subject average similarity percentage of the anterior nares´ bacterial profiles across 14 healthy children.

Child	Average similarity percentage[1]		P-Value[4]
	Within-subject[2]	Between-subject[3]	
1	33.70	26.75	0.459
2	49.30	27.22	0.009
3	45.80	16.68	0.009
4	16.10	14.22	0.545
5	54.80	28.39	0.009
6	34.60	19.69	0.009
7	41.80	27.55	0.019
8	11.90	16.26	0.009
9	48.30	24.75	0.009
10	63.40	25.45	0.009
11	37.50	26.08	0.009
12	19.00	21.15	0.382
13	24.00	21.07	0.201
14	40.50	22.46	0.069
Overall	37.19	22.69	0.015

[1]Calculation was based on PRIMER v6 software using Bray-Curtis similarity algorithm with square root transformation on phylotype level.
[2]Average similarity percentages within-subjects were calculated between bacterial profiles on three different collection days (roughly one month apart) for each child separately.
[3]Average similarity percentages between-subjects for each child were based on measurements of pairwise comparisons between all 14 children.
[4]P-values were calculated based on a Mann-Whitney-U-Test
