# Supplementary material for: Bacterial community structure and effects of picornavirus infection on the anterior nares microbiome in early childhood
Source: BMC Microbiol. 2019 Jan 7;19:1. doi: 10.1186/s12866-018-1372-8 (PMC6322332; doi:10.1186/s12866-018-1372-8)
Supplement: Supplementary file 6 — Figure S5. Relative abundance of selected genera in the anterior nares bacterial community of 12 healthy children and 12 additional children with picornavirus infection (PVI). (PDF 127 kb) [file 12866_2018_1372_MOESM6_ESM.pdf]

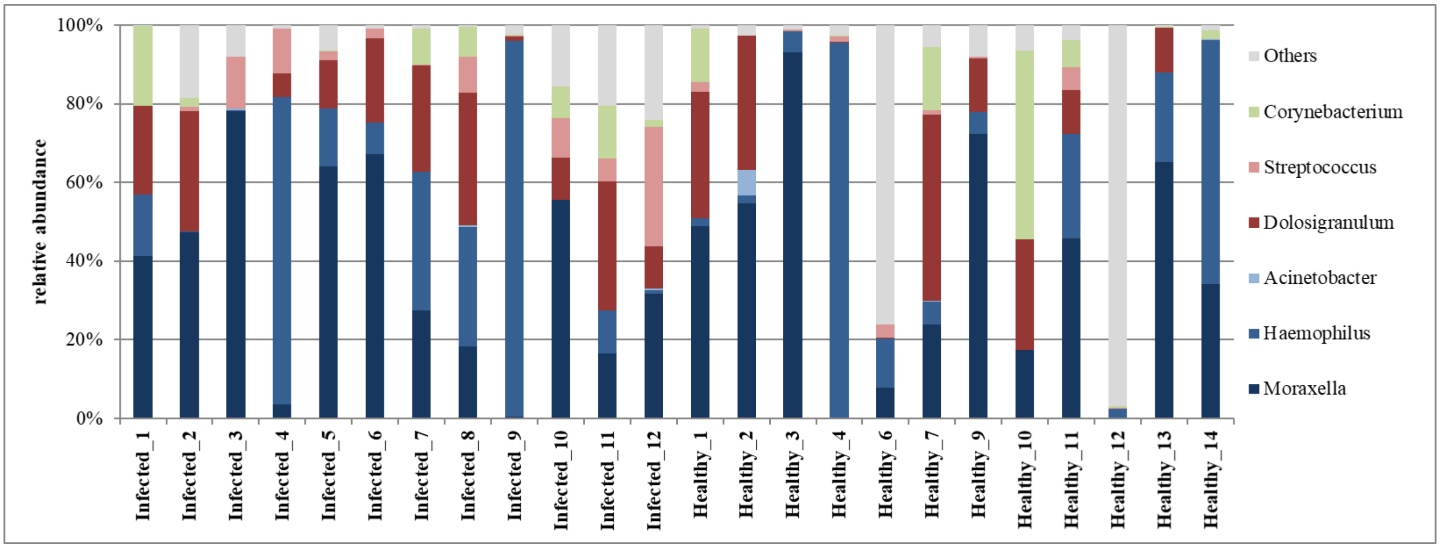

Figure S5. **Relative abundance of selected genera in the anterior nares bacterial community of twelve healthy children and twelve additional children infected with picornavirus (PV).**

Infected_1 to Infected_12: relative abundance of twelve infected children with PV; Healthy_1 to Healthy_14: relative abundance of 14 healthy children; Samples of healthy children were selected randomly out of the 42 samples (with maximum of one sample per child); Bacteria with an average relative abundance below 1% and unclassified bacteria were summarized as “Others”.
