# Supplementary material for: Bacterial community structure and effects of picornavirus infection on the anterior nares microbiome in early childhood
Source: BMC Microbiol. 2019 Jan 7;19:1. doi: 10.1186/s12866-018-1372-8 (PMC6322332; doi:10.1186/s12866-018-1372-8)
Supplement: Supplementary file 7 — Figure S6. Number and timing of nasal swab collection from 12 children with picornavirus infection (PVI). (PDF 54 kb) [file 12866_2018_1372_MOESM7_ESM.pdf]

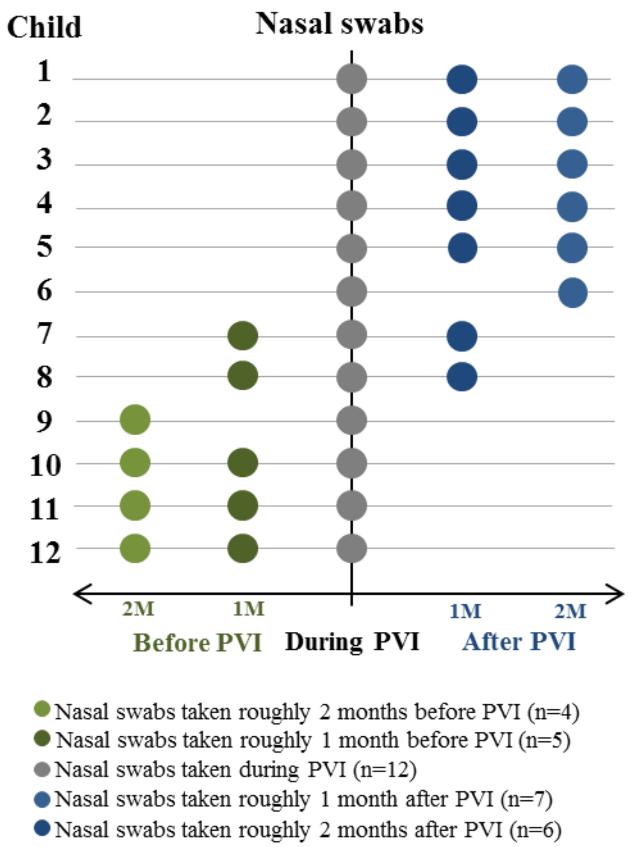

Figure S6. Number and timing of nasal swab collection from twelve children infected with picornavirus (PV). All children provided samples taken during a PV infection (PVI) (n=12); Five children provided samples taken roughly one month (1M) and two months (2M) after PVI (Child 1 to 5), and child 6 provided one swab roughly 2M after PVI; Child 7 and 8 provided samples roughly 1M before and 1M after PVI; Three children provided two samples taken roughly 1M and 2M before PVI (Child 10 to 12); One child provided one swab roughly 2M before PVI (Child 9).
