# Supplementary material for: Bacterial community structure and effects of picornavirus infection on the anterior nares microbiome in early childhood
Source: BMC Microbiol. 2019 Jan 7;19:1. doi: 10.1186/s12866-018-1372-8 (PMC6322332; doi:10.1186/s12866-018-1372-8)
Supplement: Supplementary file 8 — Figure S7. Average relative abundance of genera in the anterior nares’ bacterial communities of samples taken during and either before or after a picornavirus infection (PVI) within the same children. (PDF 98 kb) [file 12866_2018_1372_MOESM8_ESM.pdf]

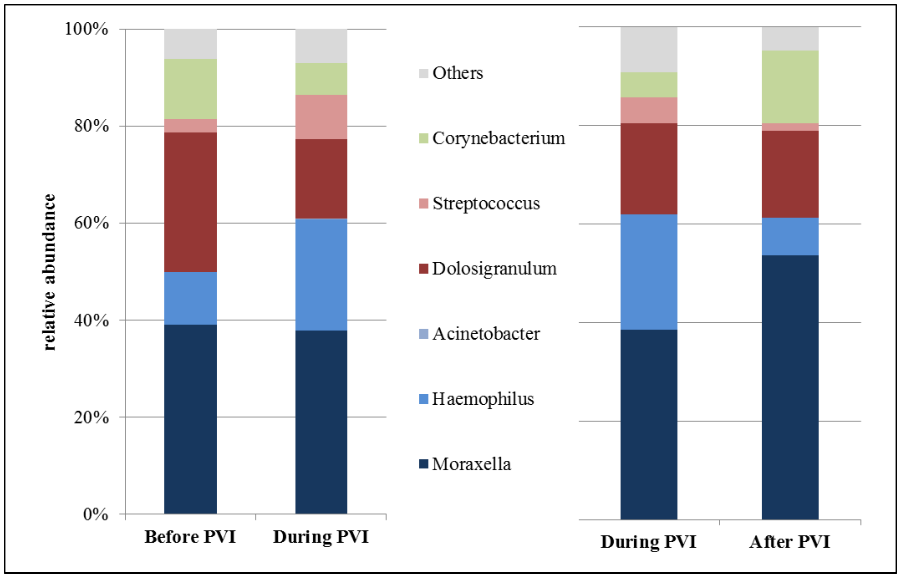

Figure S7. Average relative abundance of genera in the anterior nares´ bacterial communities of samples taken during and either before or after a picornavirus infection (PVI) within the same children.
Six children provided nasal swabs before and during PVI (five samples one month and one sample two months before PVI) (left); Eight children provided nasal swabs during and after PVI (seven samples one month after and one sample two months after PVI) (right); Bacteria with an average relative abundance below 1% and unclassified bacteria were summarized as “Others”.
