# Supplementary material for: Bacterial community structure and effects of picornavirus infection on the anterior nares microbiome in early childhood
Source: BMC Microbiol. 2019 Jan 7;19:1. doi: 10.1186/s12866-018-1372-8 (PMC6322332; doi:10.1186/s12866-018-1372-8)
Supplement: Supplementary file 9 — Figure S8. Anterior nares’ community diversity in infected children indicated by total phylotype number, Simpson index (1-Lambda), and Pielou’s evenness (J’). (PDF 229 kb) [file 12866_2018_1372_MOESM9_ESM.pdf]

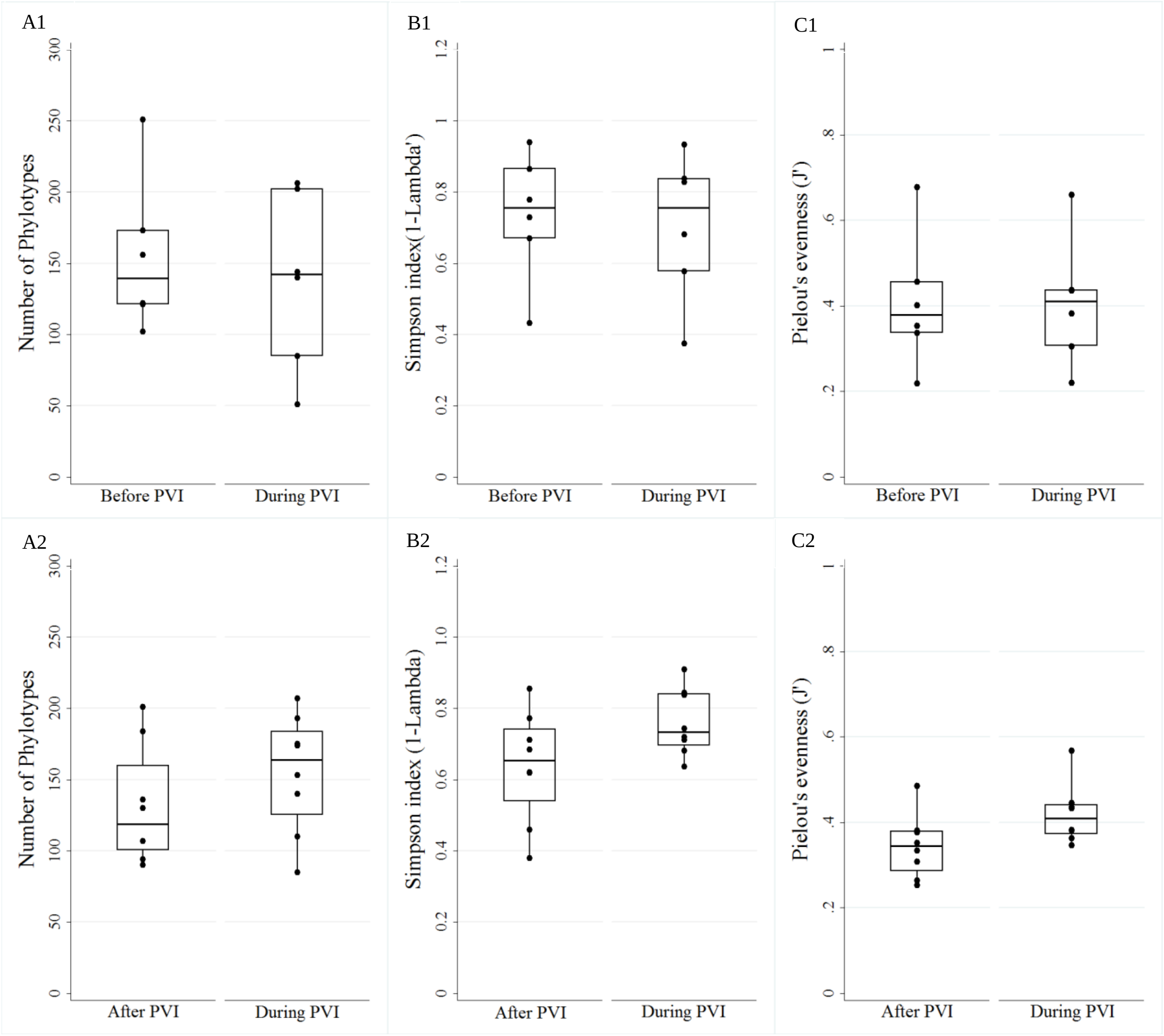

Figure S8. Anterior nares´ community diversity in children infected with picornavirus (PV) indicated by total phylotype number, Simpson index (1-Lambda), and Pielou's evenness (J').

A1 to C1: samples within the same individuals before and during PV infection (PVI) (n=6); A2 to C2: samples within the same individuals during and after PVI (n=8); A1 and A2: total number of phylotypes per child; B1 and B2: diversity calculated by Simpson index (1-Lambda); C1and C2: comparison of the evenness calculated by Pielou's evenness (J'); Values of each child are represented as one dot; Calculation was based on phylotype level using PRIMER v6 software.
