# Supplementary material for: Bacterial community structure and effects of picornavirus infection on the anterior nares microbiome in early childhood
Source: BMC Microbiol. 2019 Jan 7;19:1. doi: 10.1186/s12866-018-1372-8 (PMC6322332; doi:10.1186/s12866-018-1372-8)
Supplement: Supplementary file 10 — Table S2. Seasonal mean air temperature in Lower Saxony for winter and spring between 2010 and 2016. (PDF 31 kb) [file 12866_2018_1372_MOESM10_ESM.pdf]

Table S2. Seasonal mean air temperature in Lower Saxony for winter and spring between 2010 and 2016 (source: Climate Data Centers (CDC) of the Deutsche Wetterdienst (DWD), www.dwd.de)

Year	Mean temperature in degree Celsius	
	Winter	Spring
2010	-0.9	8
2011	0.1	10.1
2012	2.4	9.9
2013	1.2	6.6
2014	4.3	10.4
2015	2.9	8.5
2016	4.4	8.9
